# Low Magnesium Exacerbates Osteoporosis in Chronic Kidney Disease Patients with Diabetes

**DOI:** 10.1155/2015/380247

**Published:** 2015-07-27

**Authors:** Jui-Hua Huang, Fu-Chou Cheng, Hsu-Chen Wu

**Affiliations:** ^1^Department of Community Health, Chia-Yi Christian Hospital, Chiayi 600, Taiwan; ^2^Stem Cell Center, Department of Medical Research, Taichung Veterans General Hospital, Taichung 402, Taiwan; ^3^Division of Nephrology, Department of Internal Medicine, Changhua Christian Medical Foundation Erlin Christian Hospital, Changhua 526, Taiwan

## Abstract

The aim of this study is to investigate the impact of serum Mg on bone mineral metabolism in chronic kidney disease (CKD) patients with or without diabetes. A total of 56 CKD patients not receiving dialysis were recruited and divided into two groups, one group of 27 CKD patients with diabetes and another group of 29 CKD patients without diabetes. Biochemical determinations were made, and the estimated glomerular filtration rate (eGFR) was measured. Bone mineral density was measured by dual-energy X-ray absorptiometry. Serum Mg was inversely correlated with serum Ca (*P* = 0.023) and positively correlated with serum parathyroid hormone (PTH) (*P* = 0.020), alkaline phosphatase (*P* = 0.044), and phosphate (*P* = 0.040) in the CKD patients with diabetes. The CKD patients with diabetes had lower serum albumin and a higher proportion of hypomagnesemia and osteoporosis than the nondiabetic patients did (*P* < 0.05). Serum Mg was inversely correlated with eGFR in the CKD patients with or without diabetes (*P* < 0.05). Serum Mg showed an inverse correlation with 25-hydroxyvitamin D in CKD patients without diabetes (*P* = 0.006). Furthermore, the diabetic CKD patients with low serum Mg had a lower iPTH (*P* = 0.007) and a higher serum Ca/Mg ratio (*P* < 0.001) than the other CKD patients. The lower serum Mg subgroup showed a higher incidence of osteoporosis than the moderate and higher serum Mg subgroups did (66.7%, 39.4%, and 29.4%, resp.). In conclusion, low serum Mg may impact iPTH and exacerbates osteoporosis in CKD patients, particularly with diabetes.

## 1. Introduction

Osteoporosis is a skeletal disorder characterized by a low bone mass and disruption of bone architecture that leads to decreased bone strength and increased fracture risk [1]. Many factors are associated with osteoporosis, including nutritional, hormonal, and clinical factors [1, 2]. Low calcium (Ca) status is associated with a reduced bone mass and osteoporosis [3, 4]. Vitamin D deficiency impairs the absorption of Ca and leads to osteomalacia [5]. Magnesium (Mg) is also a major regulator of bone homeostasis [6]. Low Mg levels may impair the activity of parathyroid hormones (PTH) [2] and reduce serum vitamin D levels [7, 8]. In addition, chronic kidney disease (CKD) will cause abnormality of bone mineral metabolism and therefore result in complications of bone disease [9–11]. Besides, serum Mg levels may be reduced or raised with poor diabetic control or renal functional decline [12, 13], which may exacerbate bone disease [6]. However, the effect of Mg status, dietary Mg, serum Mg, and urine Mg in patients with diabetic nephropathy or CKD on Ca and bone metabolism remains unclear.

Low or high serum Mg levels may result in unwanted neuromuscular, cardiac, nervous, metabolic, or bone disorders [14, 15]. A deficit in Mg increases the risks for several diseases, including diabetes, hypertension, and cardiovascular diseases [16, 17]. Moreover, Mg deficiency is associated with low bone mass and osteoporosis [6]. Indeed, Mg deficiency affects the secretion and sensitivity of PTH. Therefore, low magnesium status reduces the activity of the 25-hydroxycholecalciferol-1-hydroxylase resulting in low serum concentrations of 1,25-dihydroxyvitamin D (1,25(OH)_2_D) and Ca [2, 7, 8]. In contrast, hypermagnesemia causes vasodilation and neuromuscular blockade [14]. Furthermore, high serum Mg levels may inhibit parathyroid hormone (PTH) secretion and also have adverse biologic effects on bone mineral metabolism [6]. However, the relationship between low or high serum Mg levels with PTH levels, vitamin D, and bone mineral metabolism remains unclear.

Chronic kidney disease causes a progressive decline in renal function over time. In the early stages, there may be no specific symptoms. Moderate-severe renal decline causes abnormality in bone and mineral metabolism, which is one of the common complications in patients with CKD [9–11]. Abnormal levels of PTH, serum vitamin D, serum phosphate (P), and serum Ca contribute to renal bone disease [9–11]. Serum Mg levels may raise with renal functional decline [12], and this eventually may be harmful to bone health [6]. In addition, diabetes is related to an increased risk of hypomagnesemia and osteoporosis [18, 19]. The patients with diabetes presented lower levels of serum Ca, vitamin D, PTH, and serum Mg [20, 21]. Furthermore, lower PTH concentration resulted in low bone formation, which may increase the risk of vertebral fractures in diabetes patients of both sexes [22]. However, there is little information about the effects of Mg on PTH, Ca, vitamin D, and bone metabolism in patients with CKD, particularly in diabetic CKD patients.

Now growing evidence shows that low Mg is associated with diabetes and nephropathy [13, 23]. Low Mg and impaired secretion and function of PTH decrease the levels of 1,25(OH)_2_D, cause low serum Ca, and are linked to bone and mineral metabolism disorders [2, 24]. Thus, low Mg may exacerbate bone disease in patients with CKD. However, it remains unclear whether moderate-severe CKD patients with diabetes still have a higher prevalence of hypomagnesemia than those of nondiabetic CKD patients because serum Mg levels may rise with renal function decline. Moreover, differences in the correlations between serum Mg with Ca, PTH, and bone mineral metabolism between CKD patients with and without diabetes have not been fully explored.

In the present study, serum Mg levels, bone mineral metabolism parameters, bone mineral density, and renal function indicators were measured. The objective of this study was to evaluate the impacts of serum Mg levels on PTH and bone mineral metabolic parameters among CKD patients with or without diabetes.

## 2. Materials and Methods

### 2.1. Study Design and Subjects

This study involved 56 stage 3–5 CKD patients not receiving dialysis who were divided into two groups as 27 CKD with diabetes and 29 CKD without diabetes. Patients were residents in a rural area and were diagnosed with chronic kidney disease at the hospital clinic of Central Taiwan. All patients were without a history of symptomatic ischaemic heart disease, heart failure, liver disease, current malignancy, and hypoparathyroidism. The study protocol was approved by the Changhua Christian Hospital Institutional Review Board (CCHIRB 090605), and informed consent was obtained from each participant.

### 2.2. Biochemical Determination

Blood samples were collected after an overnight fasting for the determinations of serum Mg, Ca, P, intact PTH (iPTH), alkaline phosphatase (ALP), 25-hydroxyvitamin D (25(OH)D), and 1,25-dihydroxyvitamin D (1,25(OH)_2_D) levels. Serum Mg levels between 1.82 and 2.31 mg/dL were defined as the normal range [25]. For patients with stages 3, 4, and 5 CKD, PTH should be maintained in the range of 35–70 pg/mL, 70–110 pg/mL, and 150–300 pg/mL, respectively [26]. For patients with stages 3 to 4 CKD, serum Ca should be maintained within normal range, 8.9–10.1 mg/dL, and serum P should be within 2.7–4.6 mg/dL [26]. For patients with stage 5 CKD, serum Ca should be 8.4–9.5 mg/dL, and serum P target should be 3.3–5.5 mg/dL [26]. For patients with stages 3 to 5 CKD, Ca-P product should be <55 mg^2^/dL^2^ [26]. The reference range of ALP is 50–136 (U/L) for laboratory used. Furthermore, vitamin D deficiency is defined as a serum 25(OH)D level of less than 20 ng/mL and vitamin D insufficiency is defined as a serum 25(OH)D level of 20 to 30 ng/mL [27]. Serum 1,25(OH)_2_D deficiency is defined as a serum 1,25(OH)_2_D level of less than 25.1 ng/mL for laboratory used.

In addition, based on the formula recommended by the Taiwan Society of Nephrology, estimated glomerular filtration rate (eGFR) was calculated as eGFR (mL/min/1.73 m2) (Simplified Modification of Diet in Renal Disease (MDRD)) = 186 × serum creatinine^−1.154^ × Age^−0.203^ in men, and 186 × serum creatinine^−1.154^ × Age^−0.203^ × 0.742 in women. The definition of chronic kidney disease (CKD) stages was based upon guidelines for the management of CKD [28]. Blood urea nitrogen (BUN) was also measured.

### 2.3. Bone Mineral Density

Bone mineral density at the left femoral neck, right femoral neck, and lumbar spine (L1–L4) was measured by dual-energy X-ray absorptiometry. Results were expressed as g/cm^2^ or as a *T*-score, which represents the number of standard deviations (SD) of the difference between a patient's BMD and that of a gender-matched young adult reference population. By definition from the World Health Organization, we have the following: (1) normal: *T*-Score at or above −1.0 SD; (2) osteopenia: *T*-Score between −1.0 and −2.5 SD; and (3) osteoporosis: *T*-Score at or below −2.5 SD [29].

### 2.4. Statistical Analysis

The Kolmogorov-Smirnov test was used to assess the normality of the distribution of investigated parameters. Continuous data were expressed (mean ± SD) and differences were tested by a 2-tailed *t*-test. Categorical data were analyzed by the Chi-square test. Continuous data of skewed distribution were presented in median, and range (25th pctl–75th pctl) and differences were examined by the Wilcoxon rank-sum test or the Kruskal-Wallis test. Multivariate analysis of the general linear model was used to analyze the association between variables. The serum Mg, bone metabolism parameters, serum Ca/Mg ratio, serum Ca × P values, and renal function indicators were log transformed before analysis because of the data's skewed distribution. Multiple regression was used to analyze all variables and presented in unstandardized coefficients (*B*), in standardized coefficients (*β*), and at a 95% confidence interval (CI) for *B*. The value *P* < 0.05 was considered statistically significant. Statistical analysis was done using SPSS 17.0 statistical software (SPSS Inc., Chicago, IL, USA).

## 3. Results

### 3.1. The Characteristics of the Subjects

The characteristics of the 56 nondialysis CKD patients with or without diabetes are shown in Table 1. The CKD patients with diabetes had significantly lower serum albumin (*P* = 0.046) and lower serum magnesium (*P* = 0.023) and osteoporosis (*P* = 0.016) when compared to those CKD patients without diabetes. In addition, age, gender, eGFR, and CKD stages were not significantly different between CKD patients with diabetes and without diabetes.

### 3.2. Relationships of Serum Mg with Renal Function and Bone Metabolism Parameters in CKD Patients with or without Diabetes

As shown in Table 2, after adjusting for confounding factors, serum Mg was inversely correlated with serum Ca (*P* = 0.015) and positively correlated with serum iPTH (*P* = 0.041) and ALP (*P* = 0.027) in the CKD patients with diabetes. Moreover, serum Mg was inversely correlated with eGFR (*P* = 0.014) and positively correlated with creatinine (*P* = 0.007) and BUN (*P* = 0.044) in the CKD patients with diabetes. However, serum Mg was not significantly associated with serum P, 25(OH)D, and 1,25(OH)_2_D in the CKD patients with diabetes. For CKD patients without diabetes, serum Mg showed an inverse correlation with 25(OH)D (*P* = 0.006). Moreover, serum Mg showed a positive correlation with serum creatinine (*P* = 0.040) and an inverse correlation with eGFR (*P* = 0.034) in the CKD patients without diabetes. There was a marginal inverse correlation between serum Mg and serum Ca in the CKD patients without diabetes (*P* = 0.065). However, serum Mg had no significant correlation with serum urea nitrogen, P, iPTH, ALP, and 1,25(OH)_2_D in the CKD patients without diabetes. On the other hand, iPTH was inversely correlated with serum Ca (*P* = 0.003) and 25(OH)D (*P* = 0.023) and positively correlated with serum ALP (*P* = 0.005) and in the CKD patients with diabetes. However, iPTH was not significantly correlated with bone metabolism parameters in CKD patients without diabetes. In addition, iPTH also was not significantly correlated with renal function indicators in the CKD patients with or without diabetes.

### 3.3. Correlation of Different Serum Mg Levels with Renal Function and Bone Metabolism Parameters

**Figure 1:**
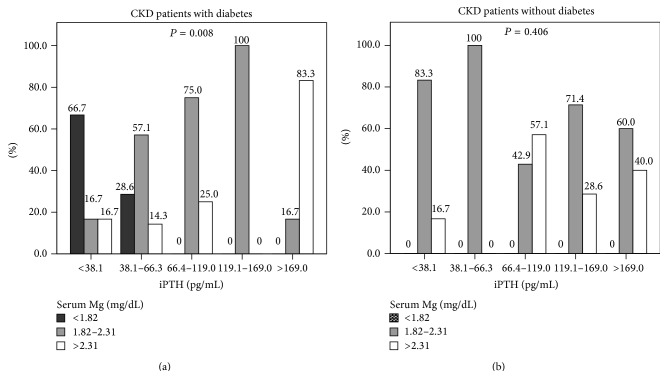
Correlation between serum Mg levels and iPTH levels. (a) CKD patients with diabetes. (b) CKD patients without diabetes. Comparisons of categorical data between two groups were analyzed by Chi-square test. When cells have expected count less than 5, data were analyzed by Fisher's Exact test. Data are presented as percentage (%). A *P* value less than 0.05 was considered statistically significant.

The correlation of different serum Mg levels and bone metabolism parameters is shown in Table 3 and Figure 1. Although serum Mg levels were not statistically significantly correlated with osteoporosis, the low serum Mg subgroup presented a higher proportion of osteoporosis than that of moderate and high serum Mg subgroups (66.7%, 39.4%, and 29.4%, resp.). Furthermore, the low serum Mg subgroup had a lower serum iPTH when compared with the moderate or high serum Mg subgroup. In contrast, the high serum Mg subgroup had a higher iPTH (*P* = 0.007), lower serum Ca (*P* = 0.018), elevated serum P (*P* = 0.026), and lower serum Ca/Mg ratio (*P* < 0.001) when compared with the moderate or low serum Mg subgroup. However, serum Mg levels were not significantly associated with serum Ca × P value, 25(OH)D, and 1,25(OH)_2_D. After stratifying the CKD patients based on presence or lack of diabetes, serum Mg levels were significantly correlated with iPTH levels in the CKD patients with diabetes (*P* = 0.008) but not in those without diabetes (Figure 1). Moreover, among six diabetic CKD patients with hypomagnesemia, four patients had low iPTH levels and four patients had osteoporosis.

### 3.4. Bone Metabolic Parameter Levels for the CKD Patients with Osteoporosis by Low and High Serum Mg Levels

As shown in Table 4, of six diabetic CKD patients with hypomagnesemia, four patients had low iPTH levels (range of 7.8–38.1 pg/mL), four patients had osteoporosis, and one patient had osteopenia. Three patients had 25(OH)D insufficiency-deficiency (range of 11.3–21.6 ng/mL), and three patients had 1,25(OH)_2_D deficiency (range of 5.1–22.1 pg/mL). Of the five CKD patients with high serum Mg and osteoporosis, four patients had diabetes and one patient did not have diabetes. The nondiabetic CKD patient with high serum Mg and osteoporosis had high iPTH levels and low levels of serum Ca, 25(OH)D, and 1,25(OH)_2_D. Furthermore, of the four diabetic CKD patients with high serum Mg and osteoporosis, three patients had also high iPTH (range of 83.8–293.0 pg/mL), 25(OH)D insufficiency-deficiency (range of 13.2–23.8 pg/mL), 1,25(OH)_2_D deficiency (range of 5.2–19.7 pg/mL), and tendency to low serum Ca (range of 8.4–9.0 mg/dL).

### 3.5. Relationships of PTH Levels with Serum Ca : Mg Ratio and Serum Ca × P Value

The relationships of PTH levels with the serum Ca/Mg ratio and serum Ca × P were analyzed via multivariate analysis using the General Linear Model, with sex, age, diabetes, and eGFR as the adjusted variables. Data are adjusted for mean and SE. The serum Ca/Mg ratio of Q1 (<38.1), Q2 (38.1–66.3), Q3 (66.4–119.0), Q4 (119.1–169.0), and Q5 (>169.0), based on quintiles of PTH levels, was 4.5 ± 0.2, 4.0 ± 0.2, 3.7 ± 0.2, 4.2 ± 0.2, and 3.5 ± 0.2, respectively (*P* = 0.019). Following Bonferroni's* post hoc* comparisons, the Q5 group had a lower serum Ca/Mg ratio than the Q1 group (*P* < 0.05). However, PTH levels were not associated with the serum Ca × P values (*P* = 0.483). In addition, linear regression analysis was performed to examine the impact of the serum Ca/Mg ratio on the PTH, also using sex, age, diabetes, and eGFR as adjusted variables. Our data indicated that the serum Ca/Mg ratio was inversely correlated with PTH levels (*P* = 0.027) and *B* was −1.455, whereas *β* was −0.305, and the 95.0% confidence interval for *B* was −2.740 to −0.170. However, PTH levels were not correlated with the serum Ca × P values (*P* = 0.182).

## 4. Discussion

Appropriate management of abnormal bone mineral metabolism may reduce CKD patients' risk of developing some complications [26]. The aim of the present study is to investigate the impacts of serum Mg levels on bone mineral metabolism in the CKD patients with and without diabetes. Our findings show that the hypomagnesemia may cause low iPTH levels and may aggravate bone mineral disorders in CKD patients with diabetes. Serum Mg levels were inversely correlated with serum Ca levels and positively correlated with iPTH, ALP, and P levels in the CKD patients with diabetes. Moreover, the CKD patients with diabetes had lower serum albumin and a higher proportion of hypomagnesemia and osteoporosis. In addition, serum Mg levels were inversely correlated with eGFR and positively correlated with serum creatinine levels in the CKD patients with or without diabetes. Serum Mg showed an inverse correlation with 25(OH)D in CKD patients without diabetes. Furthermore, serum Ca/Mg ratios were inversely correlated with the PTH levels.

### 4.1. Serum Mg and Osteoporosis in the CKD Patients with or without Diabetes

In the present study, CKD patients with diabetes had lower serum albumin and a higher proportion of hypomagnesemia and osteoporosis than those of CKD patients without diabetes. The possible explanation for this is that diabetic nephropathy is characterized by decreased renal function and significant albuminuria [30]. Serum albumin acts as a transport protein for numerous substances, including Mg, Ca, and zinc [31]. Therefore, the amount of total Mg and Ca may lower with the albumin decreasing in CKD patients with diabetes. Indeed, our data was consistent with findings from other studies [13, 32, 33]. A retrospective cohort study reported that the subjects with low serum Mg had higher proteinuria and lower serum albumin levels among CKD patients with or without diabetes. In particular, CKD patients with diabetes had more serious proteinuria and low levels of serum albumin and Mg than those of nondiabetic CKD patients [13]. Dewitte et al. also reported that renal failure patients with diabetes had lower blood Mg levels than nondiabetic patients [32]. Furthermore, hypomagnesemia is common in patients with diabetes [33], and it is associated with osteoporosis [6]. Our findings suggest that CKD patients with diabetes had lower serum albumin and a higher proportion of hypomagnesemia, and this may be related to the development of osteoporosis.

### 4.2. Serum Mg and Bone Mineral Metabolism

The differences in the relationship between serum Mg levels with bone mineral metabolism among CKD patients with and without diabetes are still under controversy. Our findings showed that serum Mg levels were positively correlated with iPTH and inversely correlated with serum Ca and 25(OH)D before stratifying the CKD patients depending on whether or not they had diabetes. After stratifying the CKD patients based on presence or absence of diabetes, there remains an inverse correlation between serum Mg and 25(OH)D in CKD patients without diabetes. Nevertheless, serum Mg levels were inversely correlated with serum Ca levels and positively correlated with iPTH and ALP in the CKD patients with diabetes but not in those without diabetes. Recently, Kanbay et al. reviewed the literature and concluded that serum Mg is inversely correlated with PTH in the general population [34]. Sakaguchi et al. reported that serum Mg levels had no correlation with serum Ca levels in CKD patients. Conversely, serum Mg levels were positively correlated with P levels [13]. Navarro et al. discovered that hypermagnesemia is common in peritoneal dialysis patients, and there was an inverse correlation between serum Mg and PTH [35]. Furthermore, the relationships of serum Mg with PTH and mineral metabolism have yielded conflicting data in hemodialysis (HD) patients. An inverse correlation between Mg levels and PTH in HD patients was claimed in some studies [36, 37]. However, another study showed that serum Mg levels positively correlated with plasma P, but no correlations between serum Mg and serum Ca or PTH in HD patients were found [38]. Our findings and data from other studies indicated that serum Mg levels may play an important role in regulating the PTH levels and bone mineral metabolism in CKD patients [13, 35]. However, relationship between serum Mg with PTH and bone mineral metabolism may vary with the presence of diabetes and different renal replacement therapies. Therefore, the effect of serum Mg levels on PTH and bone mineral metabolism in CKD patients needs further exploration.

### 4.3. Low Serum Mg Levels, Low iPTH Levels, and Osteoporosis

The most commonly encountered types of bone disease in CKD are lower turnover bone disease (adynamic bone disease), high-turnover bone disease (bone resorption), and mixed bone disease [26]. We further focus on the impacts of low or high serum Mg levels on PTH and bone mineral metabolism in CKD patients. Our data showed that serum Mg levels were correlated with iPTH levels in the CKD patients with diabetes. The subgroup with low serum Mg levels had lower iPTH levels and higher serum Ca/Mg ratios when compared with the moderate or high serum Mg levels subgroup. Moreover, our data showed six patients found hypomagnesemia in the CKD with diabetes patients but not in non-diabetes patients. Of six diabetic CKD patients with hypomagnesemia, four patients had low iPTH levels, four patients had osteoporosis, and one patient had osteopenia. Three patients had 25(OH)D insufficiency-deficiency, and three patients had 1,25(OH)_2_D deficiency. Indeed, hypomagnesemia affects the secretion and activity of PTH as well as tissue sensitivity to PTH [39, 40] and reduces the activity of the 25-hydroxycholecalciferol-1-hydroxylase, hence resulting in low serum concentrations of 1,25(OH)_2_D [2, 24]. Therefore, these diabetic CKD patients with low iPTH levels and osteoporosis may belong to lower turnover bone disease (adynamic bone disease) [26], and the low serum Mg may be a major cause. A cross-sectional study also has shown that type 2 diabetes patients have lower levels of bone resorption markers and PTH compared with subjects without diabetes [41]. Yamamoto et al. indicated that decreased PTH levels accompanied by low bone formation are related to vertebral fractures in postmenopausal women with type 2 diabetes. Therefore, lower levels of PTH may induce a lower turnover state, and this status may be correlated with the higher risk of fracture in patients with diabetes [22]. Our findings suggest that low serum Mg levels may cause insufficient PTH action, and this may eventually cause lower turnover bone disease in CKD patients with diabetes.

### 4.4. High Serum Mg, High PTH, and Osteoporosis

In contrast, our findings showed that the subgroup with high serum Mg had a higher iPTH, lower serum Ca, elevated serum P, and lower serum Ca : Mg ratio when compared with the moderate or low serum Mg subgroup. Of the five CKD patients with high serum Mg and osteoporosis, four patients have diabetes and one patient has no diabetes. The nondiabetic CKD patient with high serum Mg and osteoporosis had high iPTH levels and low levels of serum Ca, 25(OH)D, and 1,25(OH)_2_D. In addition, of the four diabetic CKD patients with high serum Mg and osteoporosis, three patients also had high iPTH, 25(OH)D insufficiency-deficiency, 1,25(OH)_2_D deficiency, and a low serum Ca trend. These CKD patients with high serum Mg levels, raised PTH levels, vitamin D deficiency, low Ca levels, and osteoporosis may be considered as having a high-turnover bone disease (bone resorption) [26]. Although high serum Mg levels may inhibit PTH secretion [6], Mg is able to reduce PTH secretion mainly when moderate to low Ca levels are present [40]. Moreover, the stimulus to produce PTH by low serum Ca levels may be more strongly influenced, rather than inhibited, by the effect of high serum Mg levels on PTH secretion [42]. Furthermore, elevated PTH levels can lead to increased bone resorption [26]. Hypermagnesemia may also cause complications in CKD patients [6]. Our data suggests that high serum Mg levels may no longer be enough to suppress PTH secretion when moderate-severe CKD patients with low serum Ca levels do not receive dialysis. Patients who maintain Ca levels within the normal range and avoid excess P and Mg levels may be key points for clinical care.

### 4.5. Serum Ca/Mg Ratio and PTH Levels

Recently, more and more studies are paying attention to the importance of Ca and Mg balance on preventing disease [43–45]. An inadequate Ca/Mg ratio may cause inflammation, cardiovascular disease, and cancer [44, 45]. However, impacts of Ca/Mg ratio on bone mineral metabolism have not been fully investigated. Our data and several studies have shown that serum Mg level was associated with serum PTH and Ca levels. Moreover, varied serum Mg and Ca levels may affect the suppression or production of PTH levels [6, 40, 42]. Therefore, interactions between serum Mg and Ca or the serum Ca/Mg ratio may be an important factor in the modulation of PTH levels and reducing the development of bone mineral disease. In the present study, we further analyze the relationship between PTH levels with serum Ca/Mg ratio and serum Ca × P values in CKD patients. Our findings showed that the serum Ca/Mg ratio was inversely correlated with the PTH levels. However, PTH levels were not associated with the serum Ca × P values. The CKD patients with low PTH levels had higher serum Ca/Mg ratios of 4.5 than those of other subgroups. In contrast, the CKD patients with high PTH levels had lower serum Ca/Mg ratios of 3.5 than those of other subgroups. In addition, CKD patients with low serum Mg and low PTH indeed had higher serum Ca/Mg ratio of 5.1. The CKD patients with high serum Mg, elevated PTH, and low serum Ca had lower serum Ca/Mg ratios of 3.4 (Table 3). Thus, we are speculating that serum Ca/Mg ratios greater than 4.5 may cause insufficient parathyroid hormone action and deteriorate lower turnover bone diseases in CKD patients. In contrast, serum Ca/Mg ratios less than 3.5 may cause stimulation to produce PTH and lead to high-turnover bone diseases in CKD patients. Our data suggest that the serum Ca/Mg ratio may be a novel determinant of PTH level, and this may be correlated with lower or high-turnover status in CKD patients. Future research shall pay much attention to the effects of serum Ca/Mg ratios on PTH levels and bone mineral metabolism as well as what the adequate serum Ca/Mg ratios in moderate-severe CKD patients are.

### 4.6. Serum Mg and Renal Functional Declines

In a recent study, serum Mg levels were inversely correlated with eGFR and positively correlated with serum creatinine levels in the CKD patients with and without diabetes. Our finding in CKD patients without diabetes was consistent with that of Sakaguchi et al., who reported that serum Mg levels had a negative correlation with creatinine clearance in patients without type 2 diabetes [13]. Dewitte et al. also found that serum Mg levels increase when creatinine clearance from 115 falls to 30 mL/min in renal failure patients without diabetes [32]. Conversely, serum Mg levels were not correlated with renal functional parameters including creatinine and GFR in the diabetic patients [13, 32]. Nevertheless, even if CKD patients with diabetes had higher proteinuria levels and lower serum Mg levels than those of nondiabetic CKD patients [13, 32], the serum Mg levels may rise when renal functional declines to moderate-severity CKD patients [12]. As the GFR falls below 30 mL/min, urinary Mg excretion may be insufficient to balance the intestinal Mg absorption [12]. Thus, the CKD patient with diabetes may be similar to the CKD patient without diabetes when declining renal function was accompanied by increases in serum Mg levels. Our data suggested that there is an inverse correlation between serum Mg levels with eGFR in the CKD patients with and without diabetes. Therefore, the dietary Mg intake should be a major determinant of serum Mg levels when the serum Mg level is raised with renal functional declines.

### 4.7. Policy Implications for Medical Care

In general, CKD disrupts Ca and P homeostasis and causes alterations of PTH and vitamin D levels [9–11]. These alterations may lead to complications, including bone and mineral metabolic diseases [9–11]. Nevertheless, the importance of Mg imbalance in disorders of bone mineral metabolism has been neglected in the clinical management of patients with CKD. The major findings of our study may have guideline implications for medical care in CKD patients, with and without diabetes. Our findings suggest that low serum Mg levels may cause insufficient parathyroid hormone action and may further lead to bone diseases in CKD patients with diabetes. For these patients, adequate Mg intake by diet or supplement may reduce the development of lower turnover bone disease. In contrast, the inhibitory effect of a high serum Mg level on PTH secretion may be offset by the stimulation produced through low serum Ca in moderate-severe CKD patients, who are not receiving dialysis. We suggest that these patients maintain a serum Ca level within the optimal range and avoid consuming excess amounts of Mg and P to reduce the risk of high-turnover bone diseases. Moreover, there should be routine monitoring of serum Mg levels, and paying attention to the balance of serum Ca and Mg is important in the assessment and management of bone mineral disorders in CKD patients with or without diabetes.

### 4.8. Limitations

Our study has several limitations. First of all, the most major one is the relatively small sample size, which may decrease the power of statistical analysis among subgroups. Inadequate statistical power may provide only pilot results for data analysis. Secondly, the generalizability of the results may be limited because the patients were residents in a rural area. Thirdly, the cross-sectional design may not conclude Mg deficiency as a cause of insufficient parathyroid hormone action in CKD patients with diabetes. Despite these limitations, our findings provide important implications for moderate-severe CKD patients. Our findings showed that low serum Mg levels may impact PTH levels and deteriorate osteoporosis. These data are inconsistent with those reported by several previous studies [2, 39]. The present data may also be applicable to nonrural CKD patients with low Mg and PTH levels. Thus, clinical staff may educate these patients with nutrition knowledge of increasing dietary Mg intakes from food or Mg supplements. They may improve the low Mg status, modulate secretion and activity of the PTH, and reduce the risk of developing osteoporosis. Further prospectively designed and supplementary studies with a large sample size may help us to reveal the effects of the Mg status on PTH and bone mineral metabolism in CKD patients with or without diabetes.

## 5. Conclusions

The CKD patients with diabetes have a higher prevalence of hypomagnesemia and osteoporosis. Low serum Mg may cause insufficient PTH action and deteriorate osteoporosis in CKD patients, particularly those with diabetes. Clinical care should focus on monitoring and managing the serum Mg levels to reduce the development of bone mineral disease in moderate-severe CKD patients who are not receiving dialysis, particularly, CKD patients with diabetes.

## Figures and Tables

**Table 1:** Characteristics of the 56 nondialysis CKD patients with or without diabetes.

Variables	Total (*n* = 56)	Diabetes (*n* = 27)	Nondiabetes (*n* = 29)	*P*
Age (y)	69.0 (58.8–75.8)	68.0 (62.0–77.0)	70 (57.0–74.5)	0.928
Gender				
Female	24 (42.9)	15 (55.6)	9 (31.0)	0.064
Male	32 (57.1)	12 (44.4)	20 (69.0)	
Estimated GFR (mL/min)	16.5 (10.5–28.8)	13.7 (9.4–29.8)	18.4 (12.0–24.8)	0.302
Chronic kidney disease				
Stage 3	13 (23.2)	7 (25.9)	6 (20.7)	0.102
Stage 4	18 (55.4)	5 (18.5)	13 (44.8)	
Stage 5	25 (44.6)	15 (55.6)	10 (34.5)	
Albumin (g/dL)	3.8 ± 0.4	3.7 ± 0.4	3.9 ± 0.4	0.046
Serum Mg (mmoL/L)				
<1.82 mg/dL low	6 (10.7)	6 (22.2)	—	0.023
1.82–2.31 mg/dL normal	33 (58.9)	13 (48.1)	20 (69.0)	
>2.31 mg/dL high	17 (30.4)	8 (29.6)	9 (31.0)	
Osteoporosis				
With	22 (39.3)	15 (55.6)	7 (24.1)	0.016
Without	34 (60.7)	12 (44.4)	22 (75.9)	

(1) eGFR, estimated glomerular filtration rate.

(2) Comparisons of continuous data between two groups were analyzed by Wilcoxon rank-sumtest. Data are median and range (25th pctl–75th pctl). The *t*-test was used for the difference in the means of two groups. Data are means ± SD.

(3) Categorical data were analyzed by the Chi-square test. When cells have expected count less than 5, data were analyzed by Fisher's Exact test. Data are number (*n*), percent (%).

(4) A *P* value less than 0.05 was considered statistically significant.

**Table 2:** Relationships between serum Mg with renal function and bone metabolism parameters in CKD patients with or without diabetes.

Dependent variables	log⁡serum Mg (mg/dL)				log⁡iPTH (pg/mL)			
	*B*	*β*	*P*	95% CI for *B*	*B*	*β*	*P*	95% CI for *B*
With diabetes								
Renal function indicators^†^								
log⁡eGFR (mL/min)	−1.616	−0.473	0.014	(−2.869, −0.363)	−0.191	−0.282	0.220	(−0.504, 0.122)
log⁡creatinine (mg/dL)	1.539	0.528	0.007	(0.461, 2.616)	0.175	0.175	0.202	(−0.101, 0.451)
log⁡BUN (mg/dL)	0.952	0.391	0.044	(0.026, 1.878)	0.027	0.056	0.810	(−0.202, 0.255)
Bone metabolism parameters^‡^								
log⁡iPTH (pg/mL)	2.296	0.454	0.041	(0.109, 4.483)				
log⁡Ca (mg/dL)	−0.134	−0.546	0.015	(−0.239, −0.028)	−0.030	−0.626	0.003	(−0.049, −0.012)
log⁡P (mg/dL)	0.272	0.312	0.106	(−0.063, 0.608)	0.040	0.231	0.222	(−0.026, 0.106)
log⁡ALP (U/L)	0.719	0.449	0.027	(0.093, 1.345)	0.168	0.529	0.005	(0.056, 0.279)
log⁡25(OH)D (ng/mL)	−0.369	−0.221	0.420	(−1.305, 0.567)	−0.188	−0.570	0.023	(−0.348, −0.029)
log⁡1,25(OH)_2_D (pg/mL)	−0.286	−0.081	0.745	(−2.094, 1.523)	−0.077	−0.110	0.649	(−0.422, 0.269)

Without diabetes								
Renal function indicators^†^								
log⁡eGFR (mL/min)	−1.091	−0.381	0.034	(−2.092, −0.090)	0.040	0.084	0.665	(−0.150, 0.231)
log⁡creatinine (mg/dL)	0.922	0.404	0.040	(0.046, 1.798)	−0.045	−0.119	0.577	(−0.210, 0.120)
log⁡BUN (mg/dL)	0.426	0.172	0.368	(−0.531, 1.382)	−0.020	−0.047	0.814	(0.814, 0.149)
Bone metabolism parameters^‡^								
log⁡iPTH (pg/mL)	1.057	0.176	0.413	(−1.566, 3.679)				
log⁡Ca (mg/dL)	−0.153	−0.407	0.068	(−0.318, 0.012)	0.007	0.118	0.597	(−0.021, 0.026)
log⁡P (mg/dL)	0.148	−0.135	0.508	(−0.605, 0.308)	−0.065	−0.355	0.065	(−0.134, −0.004)
log⁡ALP (U/L)	0.221	0.155	0.469	(−0.400, 0.841)	0.085	0.357	0.078	(−0.010, 0.179)
log⁡25(OH)D (ng/mL)	−1.250	−0.580	0.002	(−1.993, −0.507)	−0.079	−0.219	0.273	(−0.223, 0.066)
log⁡1,25(OH)_2_D (pg/mL)	0.895	0.255	0.245	(−0.656, 2.446)	0.107	0.183	0.392	(−0.147, 0.362)

(1) eGFR, estimated glomerular filtration rate; BUN, blood urine nitrogen; iPTH, intact parathyroid hormone; ALP, alkaline phosphatase; 25(OH)D, 25-hydroxyvitamin D; 1,25(OH)_2_D, 1,25-dihydroxyvitamin D.

(2) All outcomes of the multiple regression analysis are presented in unstandardized coefficients (*B*) and standardized coefficients (*β*) and at a 95% confidence interval (CI) for *B*. A *P* value less than 0.05 was considered statistically significant. ^†^Adjusted gender and age. ^‡^Adjusted gender, age, albumin, and eGFR.

**Table 3:** Correlation of different serum Mg levels with osteoporosis and bone metabolism parameters.

Variables	Serum Mg (mg/dL)			*P*
	<1.82 (*n* = 6)	1.82–2.31 (*n* = 33)	>2.31 (*n* = 17)	
Osteoporosis				
With	4 (66.7)	13 (39.4)	5 (29.4)	0.314
Without	2 (33.3)	20 (60.6)	12 (70.6)	
Bone metabolism parameters				
iPTH (pg/mL)	32.2 (20.0–62.4)	86.4 (45.9–133.0)	126.0 (80.8–221.5)	0.007
Serum Ca (mg/dL)	9.0 (8.7–9.5)	8.9 (8.6–9.3)	8.7 (8.2–8.9)	0.018
Serum P (mg/dL)	3.5 (3.1–4.1)	3.9 (3.4–4.1)	4.4 (3.7–5.0)	0.026
ALK-P (U/L)	88.5 (74.3–102.4)	79.0 (66.5–89.8)	87.0 (79.0–116.5)	0.061
25(OH)D (ng/mL)	27.2 (17.7–37.3)	22.7 (18.5–28.0)	23.6 (14.6–28.0)	0.631
1,25(OH)_2_D (pg/mL)	24.0 (15.5–29.4)	16.6 (11.9–31.2)	15.4 (11.8–23.9)	0.516
Serum Ca/Mg ratio	5.1 (5.0–5.6)	4.1 (3.8–4.4)	3.4 (2.8–3.6)	<0.001
Serum Ca × P value	31.2 (27.8–38.1)	33.8 (29.5–38.3)	37.4 (34.6–41.9)	0.128

(1) iPTH, intact parathyroid hormone; ALP, alkaline phosphatase; 25(OH)D, 25-hydroxyvitamin D; 1,25(OH)_2_D, 1,25-dihydroxyvitamin D.

(2) Comparisons of continuous data between three groups were analyzed by Kruskal Wallis test. Data are median and range (25th pctl–75th pctl).

(3) Comparisons of categorical data between two groups were analyzed by Chi-square test. When cells have expected count less than 5, data were analyzed by Fisher's Exact test. Data are number (*n*), percent (%).

(4) A *P* value less than 0.05 was considered statistically significant.

**Table 4:** Bone metabolic parameters levels for the CKD patients with osteoporosis by low and high serum Mg levels.

Case	CKD stage	iPTH (pg/mL)	Ca (mg/dL)	P (mg/dL)	25(OH)D (ng/mL)	1,25(OH)_2_D (pg/mL)	Bone health status
*Low serum Mg *							
Diabetes							
1	3	61.3	9.0	3.1	21.6	22.1	Osteoporosis
2	3	26.2	9.4	3.3	37.8	25.9	Osteoporosis
3	4	7.8	9.0	3.1	37.1	35.2	Osteoporosis
4	5	38.1	8.6	4.4	19.9	27.4	Osteoporosis
5	4	24.1	9.7	4.1	32.8	19	Osteopenia
6	3	65.8	8.7	3.6	11.3	5.1	Normal
*High serum Mg *							
Diabetes							
1	3	51.5	8.7	3.6	23.8	26.8	Osteoporosis
2	4	83.8	9.0	3.9	13.2	15.9	Osteoporosis
3	5	184.0	8.4	5.9	25.1	19.7	Osteoporosis
4	5	293.0	8.7	4.7	15.9	5.2	Osteoporosis
Nondiabetes							
1	5	169.0	8.7	4.2	17.8	3.4	Osteoporosis

(1) For patients with stages 3, 4, and 5 CKD, PTH is in the range of 35–70 pg/mL, 70–110 pg/mL, and 150–300 pg/mL, respectively.

(2) For patients with stages 3 to 4 CKD, serum Ca should be maintained within normal range, 8.9–10.1 mg/dL, and serum P should be within 2.7–4.6 mg/dL. For patients with stage 5 CKD, serum Ca should be 8.4–9.5 mg/dL and serum P target should be 3.3–5.5 mg/dL.

(3) Vitamin D deficiency is defined as a serum 25(OH)D level of less than 20 ng/mL and vitamin D insufficiency is defined as a serum 25(OH)D level of 20 to 30 ng/mL. Serum 1,25(OH)_2_D deficiency is defined as a serum 1,25(OH)_2_D level of less than 25.1 ng/mL.

## Abbreviations

ALP: Alkaline phosphatase
BUN: Blood urea nitrogen
Ca: Calcium
CKD: Chronic kidney disease
eGFR: Estimated glomerular filtration rate
HD: Hemodialysis
iPTH: Intact parathyroid hormone
MDRD: Modification of Diet in Renal Disease
Mg: Magnesium
1,25(OH)_2_D: 1,25-Dihydroxyvitamin D
25(OH)D: 25-Hydroxyvitamin D.
